# Ethnic disparities and COVID-19 pneumonia in Ireland: a single-centre descriptive study of hospitalised patients in a tertiary university teaching hospital

**DOI:** 10.1007/s11845-023-03597-y

**Published:** 2024-01-17

**Authors:** Rhea O’Regan, Finbarr Harnedy, Bearach Reynolds, Liam Cormican

**Affiliations:** Department of Respiratory Medicine, Connolly Hospital Blanchardstown, RCSI Hospital Group, Dublin, Ireland

**Keywords:** COVID-19, Ethnic minorities

## Abstract

In this study, we aim to describe the demographic, clinical and imaging characteristics, treatment course and subsequent outcomes of the first 116 cases presenting to a tertiary Dublin hospital with COVID-19 infection and to compare whether ethnic minority background was a risk factor for poorer disease outcomes in this cohort. Of 116 cases analysed, 100 (86%) patients presented from the community, 6 (5%) from care homes and 10 (9%) were existing inpatients. Fifty-four (46%) patients identified as being from an ethnic minority group. One hundred fourteen (98%) patients reported two or more symptoms at time of diagnosis with 81 (70%) patients having confirmed radiological findings of COVID-19 infection. Median duration of symptoms prior to hospital presentation was 6 days (IQR 3–10 days). The median age at presentation was 52 years (IQR 43–65). Co-morbidities recorded included hypertension, hyperlipidaemia, type 2 diabetes mellitus, underlying respiratory disease, previous or current malignancy and current smoker. Twenty-six patients (22%) required ICU admission, 20 (76.9%) of these were from all other ethnic groups combined and 6 (10%) from White Irish group. Adjusting for variables of age, ethnicity and gender, all other ethnic groups combined were five times more likely to require ICU admission than White Irish group (Table 5). Patients from all other ethnic groups combined admitted to ICU were significantly younger than patients from White Irish group (OR 50.85 vs 62.83, *P* = 0.012). Our hospital’s catchment area serves a wide-ranging and diverse population with many ethnic minority groups represented. Our data demonstrated that there was a significant overrepresentation of a younger cohort of patients from ethnic minority groups admitted to ICU with COVID-19 infection with less co-morbidities than that of the White Irish group.

## Introduction

The COVID-19 pandemic has brought attention to the significant health inequalities that exist worldwide. Similar to the 1918 Spanish influenza pandemic, it has highlighted that certain population groups are disproportionately affected compared to others [1]. Emerging evidence from studies on an international scale now demonstrate the significant differences that exist in COVID-19 morbidity and mortality rates between different population groups. This reflects the already unequal social determinants of health in our society [2]. Data from the Public Health England published in June 2020 starkly demonstrated that COVID-19 disproportionately affects ethnic minority population groups [3].

Ireland’s experience of the first wave of the COVID-19 pandemic was similar to that of the UK in highlighting health inequity. SARS-CoV-2 infection rates were highest amongst vulnerable population groups, with many outbreaks in healthcare workers, migrant workers and nursing home residents dominating media headlines [4].

Connolly Hospital serves a catchment area of approximately 330,000 people [5]. Ethnicity breakdown based on electoral divisions from the latest consensus indicate that up to 57% of this population identify as part of the majority ethnic group White Irish, with the remainder a diverse mixture of minority ethnic groups [6].

In this study, we aim to describe the demographic, clinical, imaging and laboratory characteristics, treatment course and subsequent outcomes of the first 116 cases presenting to our hospital. We describe the potential disparities in disease presentation, hospital course and outcome between a White Irish and all other ethnic groups combined population.

## Methods

### Study design and participants

We conducted a retrospective chart review of the first 116 patients that were admitted to our hospital between March 11 and May 20, 2020, with laboratory confirmed SARS-CoV-2 infection. Patients were either admitted directly from community, long-term residential care via Emergency Department or transferred from another hospital.

### Data sources and collection

Demographic characteristics, clinical features, laboratory findings, radiological manifestations, treatment course and clinical outcomes of 116 of these patients were recorded and analysed. Data was collected from patient charts, laboratory records and radiology system PACS. Information was recorded using a standardised data collection tool.

### Study outcomes

The primary outcome evaluated was patient requirement for ICU admission. Secondary outcomes evaluated included requirement for mechanical ventilation and death in White Irish group compared to all other ethnic groups combined, patients that required renal replacement therapy, LOS days in hospital and LOS days in ICU in both population groups. Outcomes were evaluated until May 22, 2020, when data collection ceased.

### Study definitions

Self-identified ethnicity was recorded from patient’s electronic care record. Terminology with respect to ethnicity is used as per most recent guidelines provided by UK government. Fever was defined as a recorded temperature of 38.0 °C or higher. Hypoxia was defined as an oxygen saturation < 94% on room air or hypoxaemia of < 7 kPa on arterial blood gas with requirement of supplemental oxygen therapy. Patients were considered to have received antibiotics during their admission if these were given for more than 24 h. Criteria for ICU admission were based on individual patient assessment. For the majority of admissions during the first wave, admission was limited to those with severe hypoxaemia of < 7 kPa, those requiring respiratory support with Fi02 of 0.6 or evidence of significant respiratory distress on examination, with need for subsequent intubation and ventilation.

### Laboratory testing

Nasopharyngeal samples were tested for SARS-CoV-2 infection by real-time reverse-transcription polymerase chain reaction (RT-PCR). Initial samples were tested at the National Virology Reference Laboratory until onsite testing was introduced.

### Statistical analysis

All univariate and multivariate analyses were conducted using STATA 16 software. Continuous variables were displayed as median values with simple or interquartile ranges as appropriate. Categorical variables were summarised as counts of all patients or a subset of evaluated patients with percentages.

## Results

A retrospective review of the first 116 patients with laboratory confirmed SARS-COV-2 infection admitted to our hospital between March 11 and May 22, 2020, was conducted. The demographic and baseline characteristics are shown in Table 1.

**Table 1:** Demographics and baseline characteristics of patients admitted with SARS-CoV-2 infection

	**All patients (*N* = 116)**	**White Irish (*N* = 62)**	**All other ethnic groups combined (*N* = 54)**	***P* value/IQR**
Age (median)	51	60	46	43–65
Female	40 (36%)	16(26%)	24 (44%)	
Male	76 (64%)	46 (74%)	30 (56%)	
Smoker	26 (22%)	19 (31%)	7 (13%)	0.02
Hypertension	40 (34%)	23 (37%)	17 (31%)	0.5
Respiratory disease	25 (22%)	18 (29%)	7 (13%)	0.04
Hyperlipidaemia	19 (16%)	14 (23%)	5 (9%)	0.05
Cerebrovascular disease	7 (6%)	6 (10%)	1 (2%)	0.07
Type 2 diabetes mellitus	17 (15%)	8 (13%)	9 (17%)	0.6
Previous/current malignancy	9(8%)	7(11%)	2(4%)	0.13
Obesity (*n* = 54)	54	19(31%)	22 (41%)	
**SARS-CoV-2 acquisition**				
Unknown	28 (24%)	15 (24%)	18 (33%)	
Travel abroad	18 (15%)	12 (19%)	1 (2%)	
Sick contacts	51 (44%)	24 (39%)	27 (50%)	
Hospital acquired/NH resident	19 (16%)	11 (17%)	8 (14%)	

The median age was 52 years old (IQR 43–65), 76 (66%) patients were male and 40 (36%) were female. Sixty-two (53%) patients identified themselves as White Irish while the remaining 54 (46%) identified as being from all other ethnic groups combined as demonstrated in Table 2. Six (5%) patients identified themselves as healthcare workers, four patients (3%) were residents of long-term care facilities and nine patients (7%) accounted for hospital acquired or nosocomial infection. Of the six healthcare workers in our cohort, all identified themselves as from all other ethnic groups combined.

**Table 2:** Ethnicity breakdown of hospitalised patients

**Ethnicity**	**All patients (*N* = 116)**	**%**
**Irish White**	62	53.45%
**Non-White Irish**	15	12.93%
**Asian**	10	8.62%
**Black**	7	6.03%
**Indian**	7	6.03%
**Roma**	15	12.93%

The most commonly recorded co-morbidities in both White Irish and all other ethnic groups combined were hypertension (34%), current or ex-smoker (22%), underlying respiratory disease (22%), hyperlipidaemia (16%) and type 2 diabetes mellitus (15%). When comparing all other ethnic groups combined with a White Irish group, these patients were less likely to have co-morbid illness (Table 1).

In terms of infection acquisition, 18 patients (15.5%) had returned from a country where there were known cases of COVID-19 pneumonia already reported. Fifty-one people (44%) were able to identify a close contact who had confirmed SARS-CoV-2 infection or were symptomatic of a respiratory illness (Table 1). Of these, 50% patients came from all other ethnic groups combined compared to 39% patients in White Irish group.

The median duration of symptoms prior to hospital presentation was 6 days (IQR 3–10 days). This was similar in both groups. The most common symptoms recorded on presentation were fever (84%), cough (83%) and dyspnoea (77%) (Table 3). There were no statistically significant differences in symptomatology reported between both groups.

**Table 3:** Clinical signs and symptoms of SARS CoV-2 infection on presentation

**Symptoms on presentation**	**All patients (*N* = 116)**	**White Irish (*N* = 62)**	**All other ethnic groups combined (*N* = 54)**	***P* value**
**Fever**	98 (84%)	49 (79%)	49 (91%)	0.17
**Cough**	96 (83%)	49 (79%)	47 (87%)	0.25
**Dyspnoea**	89 (77%)	44 (71%)	45 (83%)	0.23
**Chest pain**	21 (18%)	6 (13%)	15 (28%)	0.12
**Headache**	22 (19%)	8 (13%)	14 (26%)	0.07
**Nausea/vomiting**	36 (19%)	18(29%)	18 (33%)	0.6
**Diarrhoea**	32 (28%)	14 (23%)	18 (33%)	0.2
**Abdominal pain**	12 (10%)	7 (11%)	5 (9%)	0.72
**Myalgia/arthralgia**	45 (39%)	20 (32%)	25 (46%)	0.12
**Sore throat**	20 (17%)	11 (18%)	9 (17%)	0.55
**Fatigue**	37 (32%)	24 (39%)	13 (24%)	0.09
**Anosmia**	5 (4%)	3 (5%)	2 (4%)	0.76

**Radiological Imaging**	**All patients (*N* = 116)**	**White Irish (*N* = 62)**	**All other ethnic groups combined (*N* = 54)**	***P* value**
**Not performed**	1 (2%)	0 (0%)	1 (4%)	< 0.01
**No abnormality reported**	32 (29%)	22 (35%)	11 (21%)	< 0.01
**Unilateral infiltrates**	20 (17%)	15 (24%)	5 (9%)	< 0.01
**Bilateral infiltrates**	61 (53%)	25 (40%)	36 (67%)	< 0.01

Radiological imaging reported 67% of all other ethnic minorities combined group presented with bilateral infiltrates on chest X-ray compared to 40% of patients in White Irish group.

With respect to pharmacological interventions, 38 patients (33%) received no intervention. Seventy-eight (67%) patients received hydroxychloroquine and azithromycin or the anti-retroviral drug Kaletra (lopinavir/ritonavir) if known macrolide allergy [7]. This reflects the change in hospital guidelines that was introduced after the results of a published study highlighting the possible benefit of this drug to patients hospitalised with COVID-19 pneumonia [8]. One hundred five (91%) patients received an antimicrobial agent as per hospital protocol for presumed secondary community acquired pneumonia. Low molecular weight heparin was given to 89 (77%) patients with two patients given therapeutic dosing given high level of clinical suspicion for established thromboembolic disease. Four (3%) patients were on a novel therapeutic anticoagulant prior to admission.

In terms of non-pharmacological interventions, 98 (84%) patients required supplemental oxygen therapy by either nasal cannula or mechanical ventilation. No patients in our cohort received high flow or noninvasive ventilatory support based on national criteria as set out by HSE and Irish Thoracic Society guidelines at that time [9]. No patients received extra-corporeal membrane oxygenation (ECMO). Six patients (5%) required renal replacement therapy.

The median length of hospital stay for overall cohort of patients was 5 days (IQR 3–11). Patients in all other ethnic groups combined had a slightly longer median length of stay of 6 days compared to the White Irish group with 4.5 days.

Twenty-six patients (22%) required ICU admission, 20 (76.9%) of these were from all other ethnic groups combined and 6 (10%) from White Irish group. Adjusting for variables of age, ethnicity and gender, all other ethnic groups combined were five times more likely to require ICU admission (*P* < 0.01). Patients from all other ethnic groups combined admitted to ICU were significantly younger than patients from White Irish group (50.85 vs 62.83, *P* = 0.012).

Patients from all other ethnic groups combined had a significantly lower score than their White Irish group counterparts (2.95 vs 0.85, *P* = 0.0001). The death rate was 19% (22 patients) for this early cohort of patients (Table 4). Fifteen (13%) patients were from White Irish group and seven (6%) from all other ethnic groups combined (Table 5). At the end of our chart review, 76% (88 patients) survived to discharge. Six (5%) patients remained hospitalised. All other ethnic groups combined were more likely to be admitted to ICU during their hospital stay but death outcomes were similar in both groups.

**Table 4:** Univariate analysis of the various outcomes measures (primary and secondary) according to variable of interest (ethnic minority background)

	**All patients (*N* = 116)**	**White Irish (*N* = 62)**	**All other ethnic groups combined (*N* = 54)**	***P* value**
**Length of hospital admission (days)**	5	4.5	6	0.23
**Room air**	18 (16%)	8 (13%)	10 (19%)	0.4
**ICU admission**	26 (22%)	6 (10%)	20 (37%)	< 0.01
**Length of ICU admission (days)**	6	3 (2–12)	8 (IQR 4–11)	0.6
**Mechanical ventilation**	18 (16%)	5 (8%)	13 (24%)	0.01
**Death**	22 (19%)	15 (24%)	7 (13%)	0.3
**Discharged**	88 (76%)	44 (81%)	44 (81%)	0.3

**Table 5:** Variables associated with ICU admission as primary outcome

	Adjusted odds ratio	Confidence intervals (min–max)	*P* value
Sex (Male)	4.5	1.4–14	< 0.01
Age	1	0.99–1.08	0.1
White Irish vs all other ethnic groups combined	5	1.9–14	< 0.01
Hyperlipidaemia	0.56	0.2–0.5	0.4
Respiratory disease	0.3	0.06–1.5	0.6
Smoker	0.8	0.2–3.0	0.8

## Discussion

**Fig. 1:**
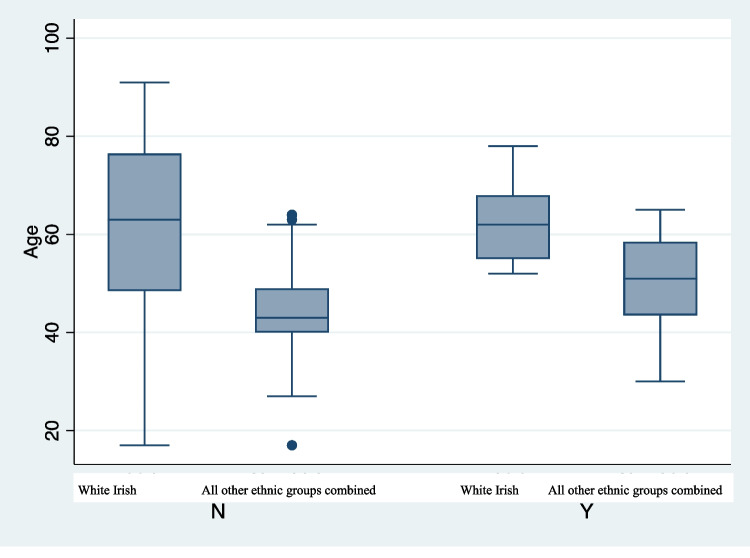
Comparing Age in White Irish vs all other ethnic groups combined admitted to ICU. N, no ICU admission; Y, ICU admission

There is a paucity of data available in Ireland on the effect the COVID pandemic has had on health outcomes in minority ethnic populations. Our study demonstrates that all other ethnic groups combined population presented at a younger age and with more severe disease than a White Irish population. They were more likely to require ICU admission and have had a longer length of hospital stay. On average, patients from all other ethnic combined group were a decade younger than patients from White Irish group in the ICU (Fig. 1). The reasons for these findings are likely complex but remain concerning. Using the Charlson Comorbidity Index as a proxy for pre-morbid conditions, we found a significantly lower score amongst our all other ethnic groups combined. It is already well established that ethnic minority groups face more difficulty in accessing healthcare services compared to majority ethnic groups [10]. Ethnic minorities are more likely to live in areas with lower HP deprivation index, live in overcrowded, urban housing and work in lower paid jobs, hence increasing risk of COVID-19 transmission [2].

Although we could not find any similar data in other Irish studies for comparison, a recent study evaluating COVID-19 outcomes in a Hispanic population in California demonstrated similar findings [11]. Dai et al. demonstrated that in their patient cohort, Hispanic patients were likely to have significantly less diagnosed co-morbidities but experience significantly worse health outcomes compared to their White counterparts. The comparability of our findings is suggestive of a problem that is not unique to Ireland, but likely to any society where minority populations may have unequal access to healthcare or face the consequences of other social determinants of health such as social exclusion or lack of appropriate education.

We found in our cohort of patients that 50% from all other ethnic groups combined population were able to identify a close contact who had been unwell. By contrast, 20% of captured data in infection acquisition in Irish population were imported cases from known affected countries.

International policy recommends continuous, cost-effective monitoring of health data to enable health services to identify and respond to health inequities as experienced by different ethnic groups [12]. There is a distinct lack of routinely collected data on social determinants and how they are reflected in the health outcomes of different ethnic minority groups in Ireland [13]. This gap in knowledge has become more and more evident as the pandemic has progressed. Our study highlights the need for greater collection of health and social care needs data for ethnic minority populations in Ireland in order to effectively mitigate the impact of COVID-19 in these settings. The impact of COVID-19 on ethnic minority populations may be viewed as a litmus test of the significant disparities that are likely to exist for other health outcomes such as respiratory and cardiovascular disease in Ireland.

There are many limitations to this study. Firstly, our sample size is small. Previous studies in other countries have demonstrated that ethnic minorities have more unfavourable morbidity and mortality outcomes from COVID-19 pneumonia [11, 14]. Similar mortality outcomes were not reflected in our data. This may partly be due to the fact that our data collection period did not capture the endpoints (death vs discharge) of all 116 patients.

We have no data available from other Irish hospital settings with which to compare our outcome measures. We were unable to accurately characterise groups based on individual ethnicity as numbers would have been too small to analyse. This may lead to individual differences in results between different ethnic minority groups. Many patients included in our study required language interpreter services. This may have led to information bias collection by healthcare workers, especially given volume of admissions during the first wave [15].

Despite its many limitations, our data captured how ethnic minorities were disproportionally affected by COVID-19 in Ireland during the first wave in an urban setting. It demonstrates the need to understand the complex patterns between health outcomes, ethnicity and the social determinants of health as applied to an Irish setting.

Our study highlights the need to inform and create health policy going forward in subsequent waves of the COVID-19 pandemic. Certain ethnic minorities may face barriers to accessing vaccination in Ireland including ongoing lack of access to healthcare, language barriers, lack of information and lack of trust in medical personnel [16]. These issues must be taken into account in public health decision-making. It is only by tackling these issues that we will improve the health of the Irish population as a whole and lessen the widening disparities already seen in other societies during the COVID-19 pandemic [1, 17].
